# Establishment of the World Health Organization 2^nd^ International Standard for Factor XI, Plasma, Human

**DOI:** 10.3389/fmed.2017.00028

**Published:** 2017-03-20

**Authors:** Helen Wilmot, Jason Hockley, Peter Rigsby, Elaine Gray

**Affiliations:** ^1^Haemostasis Section, National Institute for Biological Standards and Control, Potters Bar, Hertfordshire, UK; ^2^Biostatistics Group, National Institute for Biological Standards and Control, Potters Bar, Hertfordshire, UK

**Keywords:** International Standard for factor XI, factor XI functional activity, factor XI antigen, measurement of factor XI, multicentre study

## Abstract

The 1^st^ International Standard (IS) for blood coagulation factor XI (FXI), plasma, has been successfully used for potency labeling of FXI therapeutics and for diagnosis of FXI deficiency in patients. With stocks of the 1^st^ IS near depletion, a replacement is required. In addition to the functional activity value, assignment of an antigen value to the 2^nd^ IS would allow harmonization of antigen assay methods and differentiation of patients who have low functional activity but normal antigen FXI levels from patients who have both low functional and antigen FXI levels. The aims of this study were, therefore, to assign FXI functional activity to the 2^nd^ IS for FXI, plasma, and to additionally assign a new analyte, FXI antigen, to the same International Standard. The candidate material was prepared from double-spun, virology negative, normal plasma, which was pooled and filled into siliconized glass ampoules and subsequently freeze-dried. Assignment of the functional activity (FXI:C) value in International Units (IUs) was performed by one-stage clotting assay by 29 laboratories, relative to the 1^st^ IS. The overall geometric mean (GM) was 0.71 IU/amp with extremely low inter-laboratory variability (expressed as geometric coefficient of variation) of 1.8%. The antigen value assignment was performed by 11 laboratories and was calculated relative to normal plasma pools, as is customary with new coagulation factor analytes. The amount of antigen present in 1 ml of normal plasma was taken to be 1 U. The overall GM for the antigen assays was 0.78 IU/amp with an inter-laboratory variation of 10%. The candidate (National Institute for Biological Standards and Control code, 15/180) was established by the World Health Organization (WHO) Expert Committee on Biological Standardization in 2016 as the WHO 2^nd^ IS for blood coagulation FXI, plasma, with a functional activity value (FXI:C) of 0.71 IU/amp and an antigen value (FXI:Ag) of 0.78 IU/amp.

## Introduction

Bleeding disorders associated with factor XI (FXI) deficiency are generally mild and bleeding is most often associated with surgery or trauma, though bleeding phenotype can vary and is not always correlated to FXI coagulant activity. Deficiency is most common in Ashkenazi Jews (around 1 in 190 are homozygous for mutation in the *F11* gene and around 1 in 8 are heterozygous) but has now been identified in a wide variety of populations. There are a number of inherited mutations that cause FXI deficiency, most of which lead to a decrease in antigen and functional activity, though some patients (around 4%) experience a decrease in functional activity only (1).

The 1^st^ International Standard (IS) for blood coagulation FXI, plasma (04/102), was established by the Expert Committee on Biological Standardization (ECBS) of the World Health Organization (WHO) in October 2005 (2). The standard is used to aid diagnosis of FXI deficiency and to assign potency to licensed FXI concentrates as well as control of virus inactivated plasma products, both of which are used for treatment of patients. As with most coagulation factors and inhibitors where the International Unit (IU) is defined as the amount of activity in 1 ml of pooled normal plasma, this first IS was assigned with FXI functional activity (FXI:C) relative to local normal plasma pools only. Due to the low stock level of this International Standard, a replacement standard is required. For traceability of the established IU for FXI:C, the replacement International Standard is value assigned relative to the preceding International Standard in a multicentre collaborative study. In addition, this study also aimed to establish an antigen value for FXI (FXI:Ag) for the same candidate. This would enable differentiation between patients who have low functional activity but normal antigen FXI levels and patients who have both low functional and antigen FXI levels.

## Materials and Methods

### Candidate WHO 2^nd^ International Standard for FXI, Plasma [National Institute for Biological Standards and Control (NIBSC) Code 15/180]

Bulk material was purchased from the UK Blood Service in the form of plasma, which had been prepared by centrifugation of whole blood collected into CPD adenine anticoagulant. A second centrifugation step was performed to remove all cellular material and the plasma rapidly frozen at −70°C. Individual donations were tested at source and found to be negative for HBsAg, anti-HIV-1 and HIV-2 antibodies, and anti-HCV. The material was prepared for filling by thawing immediately prior to use at 37°C and then pooled. Glycine and HEPES were added at a final concentration of 1% w/v and 40 mM, respectively. To avoid activation of FXI by cold activation or contact with glass, plastic vessels were used and the plasma was maintained at room temperature after thawing and throughout the duration of the fill. The preparation was filled into siliconized glass ampoules and freeze-dried over a 5-day cycle in accordance with recommendations for the preparations of International Standards (3, 4). The 6,000 ampoules of 15/180 available have the following physical characteristics: mean fill mass of 1.0094 g (cv = 0.3%), mean dry weight of 0.0928 g (cv = 0.2%), mean residual moisture of 0.605% (cv = 13.7%), and mean oxygen head space of 0.23% (cv = 31.4%). The activation status of this preparation was investigated by the non-activated partial thromboplastin time, which is known to be sensitive to activated coagulation factors such as FXIa. The long mean clotting time of 300 s (*n* = 9; SD ± 2 s) for 15/180 indicates the sample to be relatively unactivated. Long-term stability of 15/180 has been assured by results from accelerated degradation study where activity of the preparations that have been stored at elevated temperature was compared with activity of the ampoules that have been stored at ultra-low temperature (−150°C). The predicted loss of FXI: C and FXI:Ag per year when the ampoules are stored at −20°C was 0.0025 and 0.039%, respectively.

### Samples in the Study

The participants were provided with the 1^st^ International Standard for FXI, Plasma (NIBSC code, 04/142), coded S and the candidate material (NIBSC code, 15/180), as coded duplicate samples A and B. In addition, local plasma pools were required to assess the relationship between the IU and local plasma unit for FXI:C and to value assign the antigen (FXI:Ag) value. The participants were asked to collect fresh local plasma pools in the study for use both fresh, coded P1, and subsequently frozen, coded P2 during the study. NIBSC in-house studies on both FXI functional activity and FXI antigen have shown that there is no significant difference in results when using fresh plasma pools compared to the same pool of plasma used after freezing; therefore, if participants were unable to collect fresh plasma pools, then frozen plasma could be used as an alternative. Across the pools used in the study (fresh and frozen) for either functional activity or antigen value, plasma from more than 20,000 donors was used.

### Collaborative Study

Of the 29 laboratories from 11 countries (Austria, Canada, Croatia, Denmark, France, Germany, Italy, The Netherlands, Spain, UK, USA) that took part in the FXI:C assignment, there were 8 clinical laboratories, 9 therapeutics producers, 6 diagnostics manufacturers, 5 regulatory laboratories, and 1 research laboratory. Eleven laboratories (2 clinical laboratories, 1 diagnostics manufacturer, 6 plasma therapeutics producers and 2 regulatory laboratories) from 8 countries (Austria, Canada, Denmark, France, Germany, Spain, UK, USA) carried out assays for FXI:Ag.

For FXI:C, participants were asked to assay coded duplicates A and B against sample S, the 1^st^ IS for FXI Plasma. Since the FXI antigen is a new analyte, the unitage of the candidate standard was assigned relative to normal plasma pools. Participants were requested to perform their in-house methods for functional and antigen measurements. All assays for FXI:C were one-stage clotting assays using activated partial thromboplastin time (APTT). In total, 13 different APTT reagents used across 14 different instrument platforms and a variety of deficient plasmas were employed by the participants and details of the reagents and deficient plasma used are indicated in Tables 1 and 2. For antigen assays, five different kits or paired antibody sets were used. The kits employed and the sources of local plasma pools used as the standard in the assays are listed in Tables 3 and 4, respectively.

**Table 1:** **Activated partial thromboplastin time (APTT) reagents used by the participants for factor XI (FXI):C assays**.

APTT reagent	Number of laboratories
Actin FS	5
Actin FSL	3
APTT-SP	5
Cephascreen	2
Cephen	1
CK Prest	4
Dapttin	1
DG-APTT Synth	1
Pathromtin SL	3
PTT A	1
Siron LS	1
SynthAFax	2
SynthASil	8

**Table 2:** **Sources of deficient plasma used by the participants for FXI:C assays**.

Source of deficient plasma	Number of laboratories
Affinity Biologicals	3
American Diagnostica	1
Dade	1
Diagnostic Grifols	2
Diagnostica Stago	8
Hematologic Technologies	2
Helena Biosciences	1
Hyphen BioMed	1
Instrumentation Laboratory	5
Precision Biologic	2
Siemens	8
Technoclone	3

**Table 3:** **Antigen kits used by the participants for FXI:Ag assays**.

Antibody kit/source	Number of laboratories
Cedarlane paired antibodies	2
Coachrom paired antibodies	2
Dunn Labortechnick kit	1
Molecular Innovation kit	1
VisuLize ELISA Kit, Affinity Biologicals	5

**Table 4:** **Plasma pools used by the participants for FXI:Ag assays**.

Plasma pool	Number of laboratories
Local, fresh, and subsequently frozen	2
Local frozen	3
Lyophilized	1
Commercial frozen	5

For both FXI:C and FXI:Ag, four independent assays for each analyte were requested. A study protocol was provided, giving guidelines on randomization of samples and sufficient replication to enable statistical analysis. Raw data were returned to the NIBSC for parallel line analysis (5) with CombiStats software (CombiStats™, Version 5.0, Council of Europe). A log_10_ transformation of the assay response was used for the analysis of the FXI antigen assays. No transformation was necessary for the FXI functional assays. All mean potencies were calculated as unweighted geometric mean (GM). Variability between assays and laboratories was expressed using geometric coefficients of variation (GCV = {10^s^ − 1} × 100% where *s* is the SD of the log_10_ transformed potency estimates). Grubbs’ Test (6) was applied to the log-transformed laboratory mean estimates in order to detect any significant outliers. Comparisons between methods were made by appropriate *t*-tests of log-transformed laboratory mean estimates. One laboratory performed single point estimates only for FXI:C, hence was excluded from the overall analysis. Where a laboratory had performed more than one set of assays using a different APTT reagent or coagulometer, these data were treated independently.

## Results and Discussion

### FXI Functional Assays

#### Analysis Relative to S, the 1^st^ IS for FXI, Plasma

**Figure 1:**
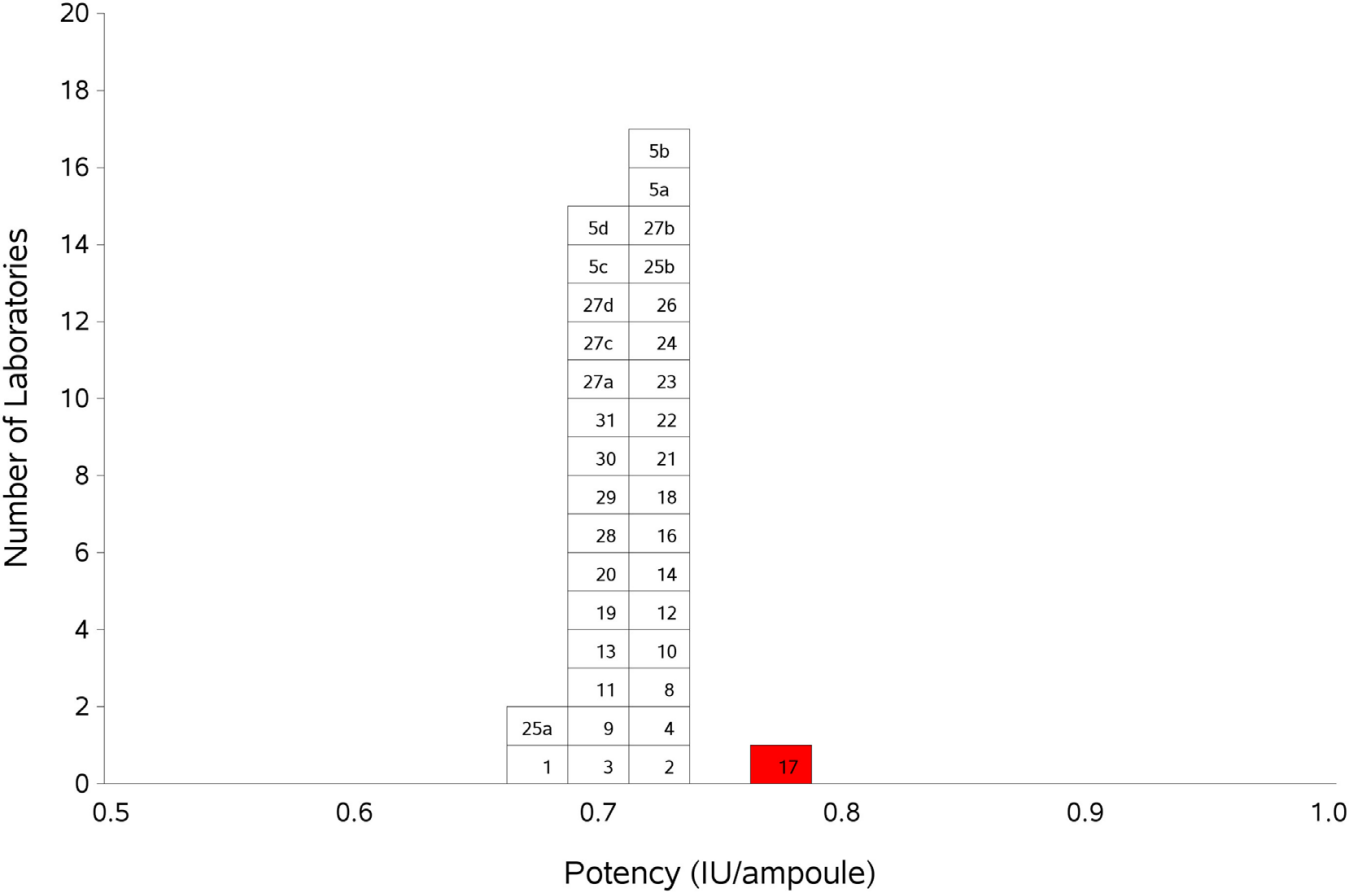
**Histogram showing each laboratory’s geometric mean for FXI:C of samples A and B combined, relative to sample S, the 1^st^ IS for FXI, Plasma**. Outliers are shown in red. The overall geometric mean was 0.71 IU/ampoule with a GCV of 1.8%.

Results for samples A and B, the coded duplicates were analyzed relative to sample S, the 1^st^ IS for FXI. The laboratories were able to perform precise assays; with intra-laboratory GCVs ranging from 0.54 to 13%, with the majority being less than 5% for both samples A or B (Table 5). The overall potency of A compared to S was 0.71 IU/amp, with a GCV of 1.60%. For B, it was also 0.71 IU/amp, with a GCV of 2.40%. The good agreement of potencies for sample A and B, the coded duplicates, showed that there was no assay design bias and that the laboratories were able to measure FXI:C accurately. As there was no significant difference between the potency estimates for samples A and B (paired *t*-test, *p* = 0.218), potencies for samples A and B were combined to give the overall GM for the candidate preparation. Each laboratory’s GM and intra-laboratory GCV are shown in Table 6. The overall result of AB vs. S was 0.71 IU/amp, the same as for A or B individually against S, and the inter-laboratory GCV was 1.80%. Results from Lab 17 were detected as statistical outliers and were excluded from the overall calculation of potency and inter-laboratory GCV. The excellent agreement between the laboratories as shown by the close distribution of laboratory GMs is demonstrated in Figure 1.

**Table 5:** **Overall geometric mean (GM) potencies, intra- and inter-laboratory variation (expressed as GCV) for FXI:C and FXI:Ag for the candidate NIBSC code: 15/180 (samples A and B)**.

	Range of intra-laboratory variation (GCV)	Inter-laboratory variation (GCV)	Overall GM in IU/amp (95% confidence limits)
FXI:C	0.54–13.0%	1.8%	0.71 (0.71–0.72)
FXI:Ag	1.1–13.0%	10.0%	0.78 (0.74–0.83)

**Table 6:** **Factor XI (FXI):C, the geometric mean (GM) for each laboratory (IU/amp) for samples A and B (coded duplicates) combined and analyzed relative to sample S, the 1^st^ IS for FXI, plasma**.

Samples AB vs. sample S		
Lab	GM (IU/amp)	GCV (%)
1	0.68	3.40
2	0.73	1.40
3	0.71	4.00
4	0.71	2.10
5a	0.72	3.20
5b	0.71	2.30
5c	0.71	2.40
5d	0.71	2.20
8	0.73	1.70
9	0.70	2.10
10	0.71	4.00
11	0.70	0.54
12	0.72	1.90
13	0.71	2.30
14	0.72	4.40
16	0.71	1.60
17	0.78	4.70
18	0.72	1.60
19	0.71	1.80
20	0.70	4.30
21	0.71	2.90
22	0.72	2.70
23	0.72	2.30
24	0.72	2.30
25a	0.67	13.0
25b	0.72	1.60
26	0.74	–
27a	0.70	–
27b	0.72	13.00
27c	0.69	12.00
27d	0.71	3.30
28	0.71	2.60
29	0.70	0.96
30	0.71	1.10
31	0.70	2.30
Overall results for AB vs. S	0.71 (0.706–0.718)	2.30
Overall results for AB vs. S, excluding outliers	0.71 (0.706–0.715)	1.80

*Intra-laboratory variation is shown as GCV (%). Lab 17 (shaded) was excluded from the overall analysis as an outlier. The overall GM and inter-laboratory GCV is also shown. Figures in brackets indicate the 95% confidence limits*.

Several of the laboratories used Actin FS, SynthASil, or APTT-SP as the APTT reagent in their assays. To determine if these reagents produced any method bias, the AB vs. S GMs of these laboratories were combined and compared to the overall GM. Table 7 shows that the results for Actin FS (0.70 IU/amp; 5 laboratories), SynthASil (0.71 IU/amp; 7 laboratories), and APTT-SP (0.72 IU/amp; 5 laboratories) compared well with the overall GM of 0.71 IU/amp for AB, indicating no method bias. The overall GM of the other APTT reagents (excluding Actin FS, SynthASil, and APTT-SP) was 0.71 IU/amp, with 95% confidence limits of 0.70–0.72 IU/amp.

**Table 7:** **Laboratory geometric means (GMs) for AB vs. S for activated partial thromboplastin time (APTT reagents) Actin FS, SynthASil, and APTT-SP, their overall GM, and comparison to overall results for AB vs. S**.

	Lab number (GM of AB vs. S, IU/amp)							Overall GM, IU/amp (95% CL)	Overall GM of AB vs. S (all APTT), IU/amp (95% CL)
Actin FS (*n* = 5)	1 (0.68)	3 (0.71)	5d (0.71)	29 (0.70)	30 (0.71)	–	–	0.70 (0.685–0.718)	0.71 (0.706–0.715)
SynthASil (*n* = 7)	2 (0.73)	10 (0.71)	11 (0.70)	20 (0.70)	22 (0.72)	23 (0.72)	31 (0.70)	0.71 (0.700–0.722)	
APTT-SP (*n* = 5)	8 (0.73)	14 (0.72)	16 (0.71)	24 (0.72)	28 (0.71)	–	–	0.72 (0.707–0.728)	

*CL, confidence limits*.

#### Analysis Relative to Plasma Pools (P)

**Figure 2:**
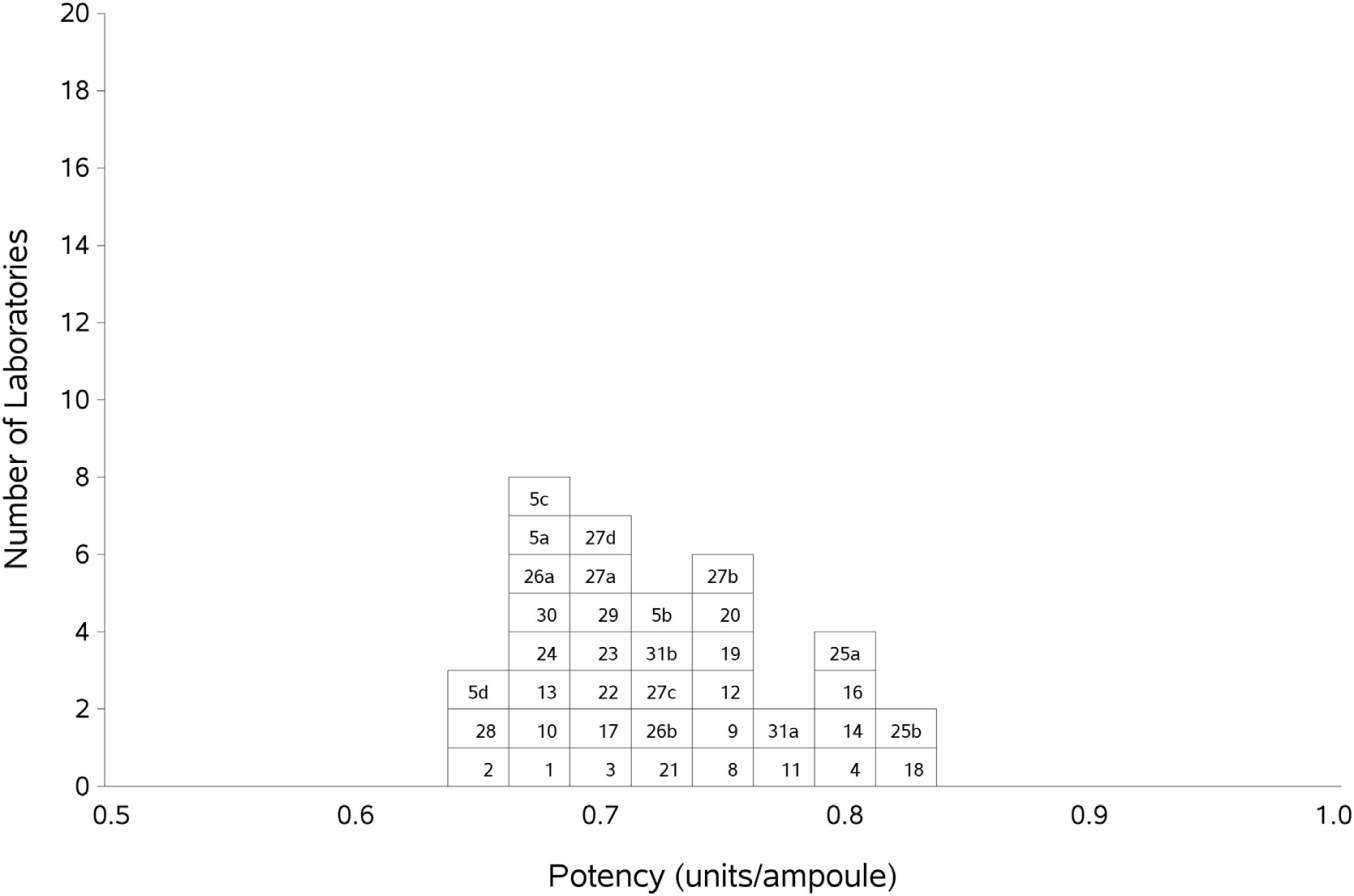
**Histogram showing each laboratory’s geometric mean for FXI:C of samples A and B combined, relative to sample P, the laboratory’s local plasma pool**. The overall geometric mean was 0.72 IU/ampoule with a GCV of 7.2%.

Participants were asked to collect two plasma pools (P1 and P2) for use both as fresh and frozen pools in the assays. Where participants were unable to collect fresh plasma pools, it was requested that two different batches of local frozen plasma pools were used. In order to assess the relationship with the plasma unit (where the amount of FXI in 1 ml of plasma is taken to be 1 U/ml), the data were analyzed relative to P. The overall GM for sample A or B relative to P was 0.72 IU/amp, agreeing well with the potency of 0.71 IU/amp relative to S, the 1^st^ International Standard for FXI, Plasma. The results for A and B relative to P were combined, and the overall results shown in Table 8 and Figure 2. The inter-laboratory variation was higher than for AB vs. S, at 7.20% overall, indicating the use of the International Standard improves inter-laboratory agreement. The overall GM for AB vs. P was 0.72 IU/amp, compared to 0.71 IU/amp relative to S. The results for AB vs. S and AB vs. P were compared using a paired two-tailed *t*-test and the result showed no significant difference between the two values (*p* = 0.215), indicating that a good relationship between the IU and the plasma unit had been maintained. A comparison of assays performed using fresh plasma pools to assays where frozen plasma pools were used showed there was no difference in the results between fresh and frozen pools (*p* = 0.704).

**Table 8:** **Factor XI:C, the geometric mean (GM) for each laboratory for samples A and B (coded duplicates) combined and analyzed relative to sample P, each laboratory’s local plasma pool**.

Samples AB vs. sample P		
Lab	GM (IU/amp)	GCV (%)
1	0.68	9.10
2	0.66	6.70
3	0.70	4.20
4	0.79	7.60
5a	0.67	1.80
5b	0.72	2.50
5c	0.66	2.50
5d	0.65	2.10
8	0.75	3.60
9	0.75	10.00
10	0.66	4.30
11	0.78	5.00
12	0.74	1.60
13	0.67	6.10
14	0.80	2.20
16	0.80	5.70
17	0.71	1.80
18	0.84	5.40
19	0.74	3.10
20	0.76	4.70
21	0.71	4.70
22	0.70	0.91
23	0.70	3.60
24	0.68	3.20
25a	0.81	–
25b	0.83	3.60
26a	0.67	–
26b	0.72	3.80
27a	0.71	2.10
27b	0.75	–
27c	0.72	12.00
27d	0.71	3.60
28	0.66	3.90
29	0.71	4.70
30	0.68	5.30
31a	0.77	1.40
31b	0.74	1.90
Overall results for AB vs. P	0.72 (0.705–0.739)	7.20

*Intra-laboratory variation is shown as GCV (%). The overall GM and inter-laboratory GCV for AB vs. P is also shown. Figures in brackets indicate the 95% confidence limits*.

### FXI Antigen Assays

Participants were asked to collect two plasma pools (P1 and P2) for use both as fresh and frozen pools in the assays. Where participants were unable to collect fresh plasma pools, it was requested that two different batches of local frozen plasma pools were used. As there is no current International Standard for FXI antigen, the units of FXI antigen in 1 ml of plasma pool was taken to be one, and the potencies of A and B calculated relative to the laboratories’ local plasma pool. Overall, the intra-laboratory variations ranged between 0.5 and 18%, with most laboratories having a GCV of less than 10% (Table 5). The variation of these assays would be expected to be higher than that of the FXI functional assays by virtue of the fact that the calculations are relative to the local plasma pools, which themselves have inherent variability. Due to the low number of assays performed using fresh plasma pools (5), it was not possible to compare fresh and frozen plasma pools.

**Figure 3:**
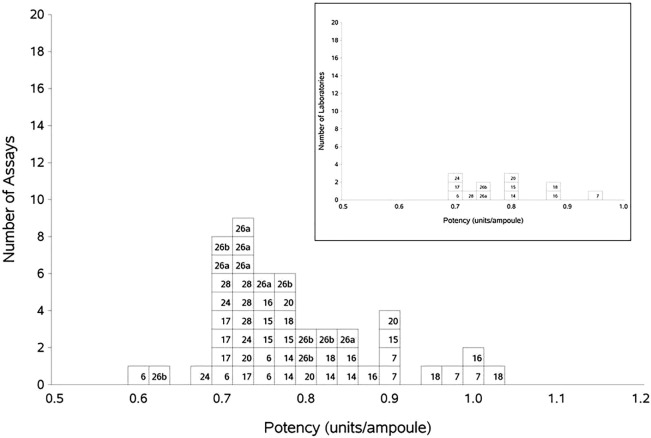
**Histogram showing each laboratory’s individual assay results for FXI:Ag of samples A and B combined, relative to sample P, the laboratory’s local plasma pool**. The overall geometric mean was 0.78 U/ampoule with a GCV of 10.0%. The inset shows each laboratory’s geometric mean for FXI:Ag samples A and B combined, relative to the laboratory’s local plasma pool (sample P).

Since A and B were coded duplicates and paired *t*-test showed that there was no significant difference between the estimates for A and B (*p* = 0.198), the results were combined to give an overall result for AB against P. The overall GM was 0.78 IU/amp, with an acceptable inter-laboratory GCV of 10%. No statistical outliers were detected. The individual assay results from each lab and the laboratory GMs are shown in Table 9 and graphically in Figure 3.

**Table 9:** **Factor XI:Ag, the geometric mean (GM) for each laboratory for samples A and B (coded duplicates) combined and analyzed relative to sample P, each laboratory’s local plasma pool**.

Samples AB vs. sample P		
Lab	GM (IU/amp)	GCV (%)
6	0.70	11.00
7	0.94	5.80
14	0.81	4.50
15	0.79	9.70
16	0.86	13.00
17	0.71	2.80
18	0.89	13.00
20	0.80	9.40
24	0.71	3.80
26a	0.74	7.60
26b	0.76	11.00
28	0.72	1.10
Overall result for AB vs. P	0.78 (0.735–0.832)	10.00

*Intra-laboratory variation is shown as GCV (%). The overall GM and inter-laboratory GCV for AB vs. P is also shown. Figures in brackets indicate the 95% confidence limits*.

Of the 12 laboratories that took part, 5 used an antigen kit from Affinity Biologicals. The GMs for AB against P of these laboratories were combined and resulted in a GM of 0.72 IU/amp (with 95% confidence limits of 0.70–0.75 IU/amp). This is slightly lower than the overall result for AB against P (0.78 IU/amp). The result for the other laboratories not using the Affinity Biologicals kit was 0.82 IU/amp, with 95% confidence limits of 0.75–0.90 IU/amp, which overlap the overall GM of AB vs. P, but not that of the laboratories using the Affinity Biologicals kit. A comparison of the two groupings was made using the unpaired two-tailed *t*-test and the difference was found to be significant (*p* < 0.05). The difference suggests that the Affinity Biologicals kit may yield lower results than other kits; however, this could also be a reflection of the different plasma pools used within each laboratory. Due to the small number of laboratories taking part in the study and the fact that the two groupings had 95% confidence limits that were extremely close to being overlapping, the consensus GM was taken as the antigen value for the candidate preparation. Any effect of minor method bias on the results is likely to be small, especially for measurement around the normal range. For measurements of abnormal samples, local qualification and validation of antigen method are advisable.

Based on the results of this study, the participants of the study and the Experts nominated by the Scientific and Standardization Committee (SSC) of the International Society on Thrombosis and Haemostasis (ISTH) agreed that the candidate preparation, NIBSC code: 15/180 is a suitable International Standard for FXI:C and FXI:Ag. In October 2016, the ECBS of the WHO established NIBSC code: 15/180 as the 2^nd^ International Standard for FXI, plasma with assigned values of 0.71 and 0.78 IU/amp for FXI functional activity (FXI:C) and FXI antigen (FXI:Ag) respectively.

## Author Contributions

HW and EG designed, organized, and wrote the manuscript. JH and PR designed and analyzed the study data.

## Conflict of Interest Statement

The authors declare that the research was conducted in the absence of any commercial or financial relationships that could be construed as a potential conflict of interest.
